# Mantle margin morphogenesis in *Nodipecten nodosus* (Mollusca: Bivalvia): new insights into the development and the roles of bivalve pallial folds

**DOI:** 10.1186/s12861-015-0074-9

**Published:** 2015-05-28

**Authors:** Jorge A. Audino, José Eduardo A. R. Marian, Andreas Wanninger, Sônia G. B. C. Lopes

**Affiliations:** Department of Zoology, University of São Paulo, Rua do Matão, Travessa 14, n. 101, 05508-090 São Paulo, SP Brazil; Department of Integrative Zoology, University of Vienna, UZA1 Althanstraße 14, 1090 Vienna, Austria

**Keywords:** Integrative microscopy, Larvae, Ontogeny, Pectinidae, Periostracal groove

## Abstract

**Background:**

Despite extensive knowledge on bivalve anatomy and development, the formation and differentiation of the mantle margin and its associated organs remain largely unclear. Bivalves from the family Pectinidae (scallops) are particularly promising to cast some light on these issues, because they exhibit a complex mantle margin and their developmental stages are easily obtained from scallop farms. We investigated the mantle margin of the scallop *Nodipecten nodosus* (L. 1758) during larval and postmetamorphic development.

**Methods:**

A thorough analysis of the mantle margin development in *Nodipecten nodosus*, from veliger larvae to mature adults, was conducted by means of integrative microscopy techniques, i.e., light, electron, and confocal microscopy.

**Results:**

Initially unfolded, the pallial margin is divided into distal and proximal regions by the periostracum-forming zone. The emergence of the pallial musculature and its neural innervation are crucial steps during bivalve larval development. By the late pediveliger stage, the margin becomes folded, resulting in a bilobed condition (i.e., outer and inner folds), a periostracal groove, and the development of different types of cilia. After metamorphosis, a second outgrowth process is responsible for emergence of the middle mantle fold from the outer surface of the inner fold. Once the three-folded condition is established, the general adult features are rapidly formed.

**Conclusions:**

Our data show that the middle mantle fold forms from the outer surface of the inner fold after metamorphosis and that the initial unfolded mantle margin may represent a common condition among bivalves. The first outgrowth process, which gives rise to the outer and inner folds, and the emergence of the pallial musculature and innervation occur during larval stages, highlighting the importance of the larval period for mantle margin morphogenesis in Bivalvia.

## Background

The molluscan mantle margin, which corresponds to the free portion of the mantle, exhibits great diversity of form and function. In bivalves, the mantle is a membranous organ consisting of left and right lobes, united dorsomedially by an isthmus that lines the interior of the shell valves and surrounds the mantle cavity [1]. The mantle margin is the free edge of this organ and bears tissue extensions named mantle (or pallial) folds, which can bear muscular, sensory or secretory structures [1]. The presence of three mantle folds in this region is claimed to be the general condition for conchiferan molluscs; nevertheless, structural diversity in the pallial margin is very pronounced among and within molluscan classes [2]. Although gastropods and scaphopods most often display a simple, swollen projection in the mantle rim, in a few gastropods the mantle margin is molded into distinctive folds [2, 3]. The monoplacophoran *Neopilina galathea* displays three folds in the mantle margin, and a periostracal groove is placed between the outer and middle folds [4]. Similarly, among living cephalopods, the mantle of *Nautilus pompilius* also exhibits the three-folded pattern [5].

In Bivalvia, the mantle margin is divided into three pallial folds, each with a specific function: the secretory outer fold, the sensory middle fold, and the muscular inner fold [6, 7]. The periostracum is formed in a deep groove between the outer and middle folds, while the shell layers are secreted by the outer mantle epithelium [8, 9]. However, exceptions to this three-folded pattern do exist, e.g., four pallial folds have been described for the Veneridae [10–12], duplication in the middle fold may occur in Donacidae [13], and two pallial folds may be present in some Arcidae, with the outer one being duplicated [14, 15].

The bivalve mantle margin displays several adaptive traits associated with the different bivalve lifestyles, resulting in the huge morphological diversity observed within the class. Different levels of fusion during mantle margin and siphon formation are among the key features associated with the evolutionary radiation of infaunal bivalves [6, 16, 17]. Specialized secretory glands from the mantle margin are widespread in many groups performing a variety of roles, e.g., cleansing, adhesion, lubrication, and boring [18–20]. Pallial tentacular structures are also present in some families, such as Limidae, Pectinidae, and Galeommatidae, performing sensorial, defensive, and secretory functions [21–23]. In addition, photoreceptors and pallial eyes have evolved independently in the mantle margin of several distantly related bivalve taxa [24, 25]. Even though extensive amount of knowledge concerning the morphology of the bivalve mantle margin has been produced, these studies have mainly focused on adult anatomy, important developmental and functional issues remaining largely unclear [26].

Bivalve organogenesis is poorly understood compared to other molluscan groups such as gastropods, and further investigations applying techniques of microanatomy are vital to evaluate the functional ontogeny in the Bivalvia [27]. The development of organs and structures, such as gills, foot, velum, and, most of all, the shell, has been thoroughly documented for several bivalve representatives (e.g. [28–34]). Apart from the detailed study on mantle anatomy of *Ostrea edulis* larvae [35], details on the larval mantle are fragmentary and restricted to descriptions on general larval morphology [36–40]. As a consequence, several questions on bivalve mantle morphogenesis remain unanswered. For instance, a developmental hypothesis assuming the origin of the middle mantle fold from the inner fold has been proposed based on anatomical features of the mantle margin in some adult bivalves [15]. However, further developmental evidence is still necessary to test this hypothesis and understand the changes and mechanisms underlying mantle fold differentiation.

Scallops (bivalves of the family Pectinidae) are particularly promising to cast some light on the origin and differentiation of the bivalve mantle margin. Due to their economical importance, developmental stages are easily obtained from scallop farms. In addition, pectinids bear an especially complex mantle margin, displaying distinct organs and pallial structures, such as elaborate eyes and tentacles. Scallops are a well-studied group and large literature on their behavior, taxonomy, phylogeny, and aquaculture is available (e.g. [41–44]). Their pallial eyes have been extensively studied due to their intricate structure and optical performance (e.g. [45–49]). The inner mantle fold is a muscular curtain responsible for the regulation of the water flow into and out of the mantle cavity, especially during clapping movements and swimming behavior [50–52]. Although there is a plethora of studies on scallop pallial structures, there is no specific information concerning the anatomy and developmental changes of their mantle margin.

The present study aims at analyzing the morphogenesis of the mantle margin in the scallop *Nodipecten nodosus* (Linnaeus, 1758). We provide a thorough description of this region throughout its development, from veliger larvae to mature adults, by means of light, electron, and confocal microscopy.

### Orientation and axis determination

Larval orientation is herein defined in accordance with comparative lophotrochozoan larval anatomy, i.e., with anterior corresponding to the position of the apical tuft, posterior corresponding to the opposite region, and ventral being defined by the position of the foot. In postmetamorphic bivalves, morphological axis designation is commonly such that the mouth position defines anterior and the hinge line dorsal (with the opposite as ventral). These terms are used herein, although we are aware of the semantic issues concerned with larval versus adult axis designations.

## Results

**Fig. 1:**
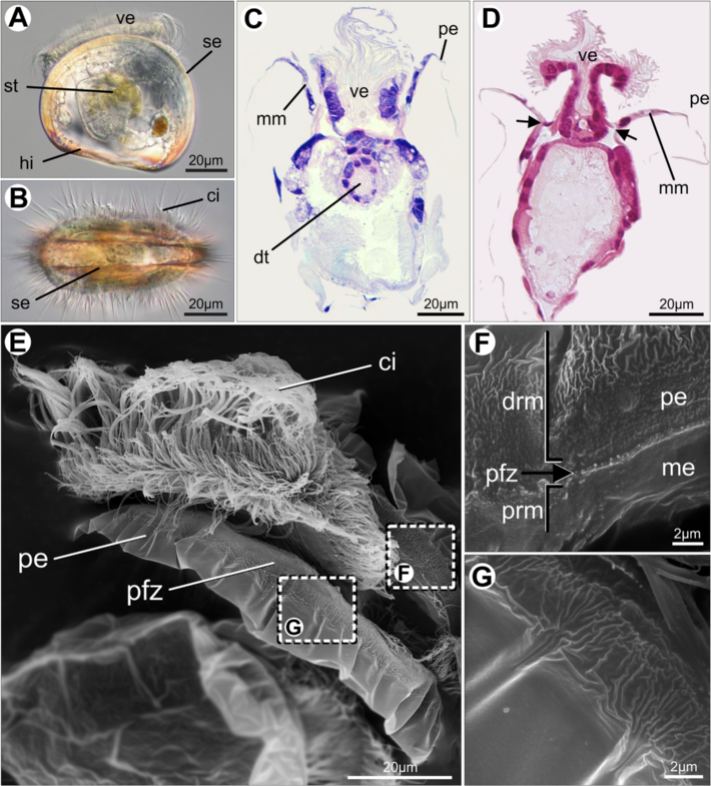
Veliger larvae of *Nodipecten nodosus*. **a** Specimen observed by light microscopy applying differential interference contrast; lateral view, anterior to the top, ventral to the right. **b** Same individual, anterior view, ventral to the right. **c** Cross section of a veliger with the larval velum retracted (anterior to the top). Toluidine blue and basic fuchsin. **d** Cross section of a veliger with the larval velum exposed. Arrows point to the membrane connecting the larval velum and the unfolded mantle margin. Hematoxylin and eosin. **e** General morphology of a decalcified veliger, scanning electron micrograph (SEM); ventral view, anterior to the top. **f** Detail of the periostracum-forming zone (arrow), as indicated in the inset of **e**, defining the distal and proximal regions of the mantle margin. Lateral view. **g** Detail of the newly formed periostracum with smooth and wrinkled zones, as indicated in the inset of **e**. Lateral view. Abbreviations: ci, cilia from the larval velum; drm, distal region of the mantle margin; dt, digestive tract; hi, hinge line; me, mantle epithelium; mm, mantle margin; pe, periostracum; pfz, periostracum-forming zone; prm, proximal region of the mantle margin; se, shell edge; st, stomach; ve, larval velum

**Fig. 2:**
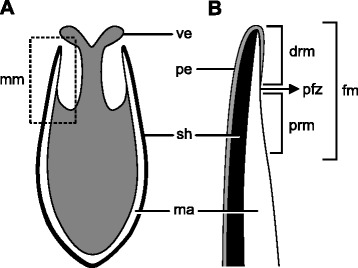
Schematic representation of the mantle margin in veliger larvae of *Nodipecten nodosus*. **a** Schematic representation of a cross-section through the larval body, anterior region to the top. **b** The unfolded mantle margin, framed in **a**, is divided into two regions, the distal and proximal ones, by the periostracum-forming zone. Abbreviations: drm, distal region of the mantle margin; fm, free mantle margin; ma, mantle; mm, mantle margin; sh, shell; pe, periostracum; pfz, periostracum-forming zone; prm, proximal region of the mantle margin; ve, larval velum

In veliger larvae of *Nodipecten nodosus* (Fig. 1a, b), the mantle comprises two lobes that enclose the body of the animal and define a small pallial cavity where the larval velum is located (Fig. 1c, d). The mantle margin runs alongside the shell edge from each side of the mantle isthmus (posterior region where the hinge is situated). The pallial margin of live larvae cannot be properly visualized by means of conventional light microscopy because of its transparency and location underneath the shell edge, being also partly obliterated by the large, antero-ventrally located ciliated velum (Fig. 1a, b). Histological cross sections from decalcified veligers show the mantle margin as a slender mantle projection connected to the velum by a short membrane (Fig. 1c, d). A schematic illustration of the larval mantle margin is represented in Fig. 2. The margin, which becomes thinner towards the distal region, is unfolded, and no particular structure is present. The periostracum is a fine layer originating in the inner epithelium of the pallial margin, close to the distal region (Fig. 1e, f, 2). The secretion occurs in a well-defined site, the periostracum-forming zone (PFZ), marked by a line parallel to the margin between the mantle epithelium and the newly formed periostracum sheath (Fig. 1f). From this region, the periostracum begins as a wrinkled band, but becomes smooth when covering the rest of the distal mantle margin (Fig. 1g). In the proximal region, immediately before the PFZ, the exposed inner epithelium contains short microvilli on its surface; no cilia are present (Fig. 1f).

**Fig. 3:**
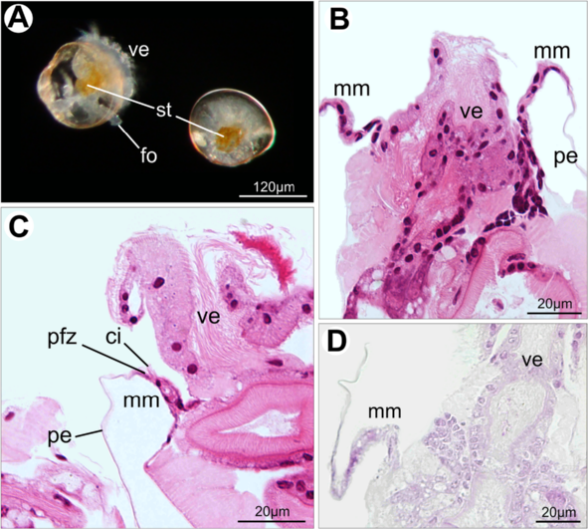
Pediveliger larvae of *Nodipecten nodosus*. **a** Light with differential interference contrast; lateral view, anterior to the top in the right specimen and to the slightly top right in the left specimen. **b** Cross section of the mantle margin and velum in a specimen younger than **c**. Anterior to the top. Hematoxylin and eosin. (HE). **c** Cross section of the mantle margin showing cilia close to the periostracum-forming zone. HE. **d** Cross section of an individual showing no particular reaction to periodic acid-Schiff staining (PAS). Abbreviations: ci, cilia; fo, foot; mm, mantle margin; pe, periostracum; pfz, periostracum-forming zone; st, stomach; ve, larval velum

**Fig. 4:**
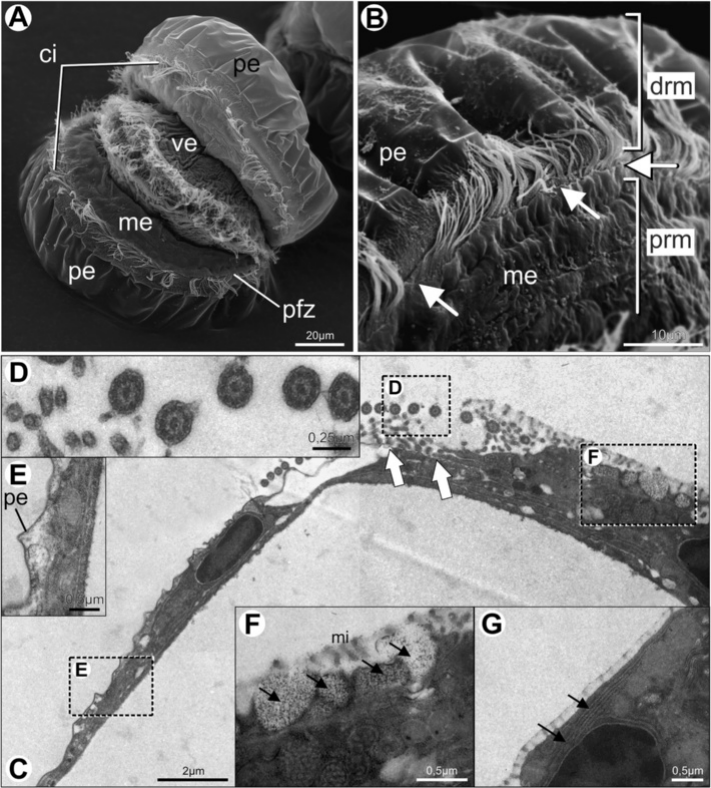
Pediveliger larvae of *Nodipecten nodosus.* Scanning electron micrographs (**a**–**b**) and transmission electron micrographs (**c**–**g**), respectively. **a** General morphology of the mantle margins exposed by the retracted larval velum; anterior view, ventral to the right. **b** Detail of the mantle margin showing the periostracum-forming zone (arrows) and the adjacent row of cilia; lateral view, anterior to the top. **c** Mantle margin of a pediveliger larva, proximal region to the right and distal region to the left; white arrows point to sites of periostracum secretion. **d** Cross section of cilia present in the periostracum-forming zone showing the classical microtubular arrangement “9 + 2”. **e** Periostracal layer covering the distal region of the mantle. **f** Detail of electron-lucent vesicles (black arrows) present in the inner epithelium of the proximal region of the mantle, covered by short microvilli (**g**) Detail of the rough endoplasmic reticulum around the nucleus of a cell from the proximal region of the mantle margin. Abbreviations: ci, cilia; drm, distal region of the mantle margin; me, mantle epithelium, mi, microvilli; pe, periostracum; pfz, periostracum-forming zone; prm, proximal region of the mantle margin; ve, larval velum

By the early pediveliger stage, larvae of *Nodipecten nodosus* grow and develop an extensible foot (Fig. 3a). The swimming habit prevails, although the crawling behavior becomes gradually common upon available hard surfaces. The general morphology of the pallial margin in pediveligers closely resembles that observed in veligers, including an unfolded projection (Fig. 3b). However, a major difference is present in both mantle margins: the emergence of a row of cilia along the entire periostracum-forming zone (Figs. 3c, 4a, b). These cilia exhibit the classical 9 + 2 microtubular arrangement (Fig. 4c, d). From the PFZ, the periostracum covers the rest of the inner distal pallial margin (Fig. 4c, e) and extends to cover the shell. The proximal portion of the mantle margin is characterized by the presence of several secretory cells, particularly on the inner epithelium (Fig. 4c, f), as well as well-developed rough endoplasmic reticulum around the cell nuclei (Fig. 4c, g). The content of those vesicles are granular, electron-lucent and seems to be released on the inner surface (Fig. 4f). Despite the presence of secretory activity in this region, no evidence for mucopolysaccharides was detected applying standard histochemical techniques (Fig. 3d).

**Fig. 5:**
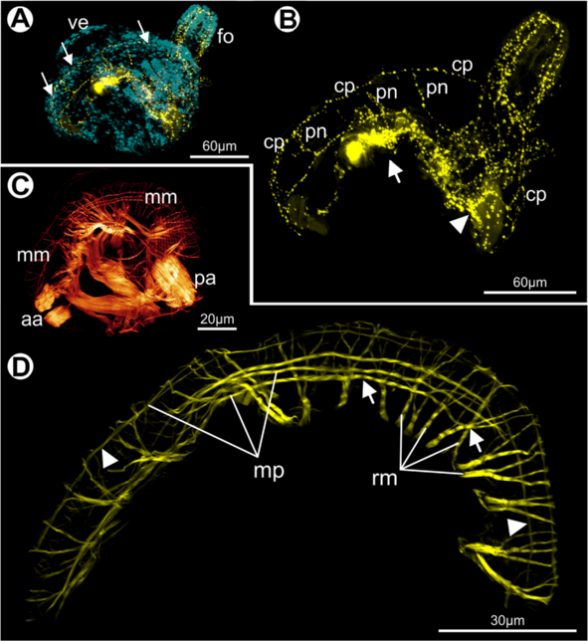
Immunocytochemistry in pediveliger larvae of *Nodipecten nodosus.* Lateral view (anterior to the top, ventral to the right). Staining with anti-serotonin (**a**–**b**) and phalloidin (**c**–**d**), as revealed by confocal laser scanning microscopy (CLSM). **a** Pediveliger showing strong serotonin-like immunoreactivity (yellow) in the central and peripheral nervous system, particularly in the circumpallial nerve (arrows) and the pedal nervous plexus. Nuclei are stained in blue by DAPI. **b** 3D reconstruction of the pediveliger nervous system based on a CLSM image stack showing the mantle innervation in detail. Arrow points to the developing cerebral ganglia and arrowhead to the visceral ganglion. **c** Larval musculature of the right half of an individual. **d** 3D reconstruction of the pallial musculature, showing retractor and margin-parallel muscles; arrows point to striated fibers and arrowheads to suggested non-striated fibers. Abbreviations: aa, anterior adductor; cp, circumpallial nerve; fo, foot; mm, mantle margin; mp, margin-parallel muscles; pa, posterior adductor; pn, pallial nerves; rm, retractor muscles; ve, larval velum

Although the mantle margin of the pediveliger is quite similar to that of the veliger, crucial anatomical changes arise during this period, contributing to the morphogenesis of the pallial system. In scallop veligers, no muscles or nerves were detected associated with the mantle margin (data not shown). In contrast, distinct serotonergic fibers are found in the mantle of pediveligers, where a conspicuous nerve runs along the entire edge (Fig. 5a), likely corresponding to the developing circumpallial nerve. While the mantle is dorsally innervated by projections from the apical-cerebral ganglia, the ventral portion receives fibers from the visceral ganglion (Fig. 5b). A distinct pallial musculature is also developed in pediveliger larvae, containing smooth and striated myofibers (Fig. 5c, d). While some of them are parallel to the edge, other bundles are branching retractors, attached to the shell, that run towards the mantle margin (Fig. 5d).

**Fig. 6:**
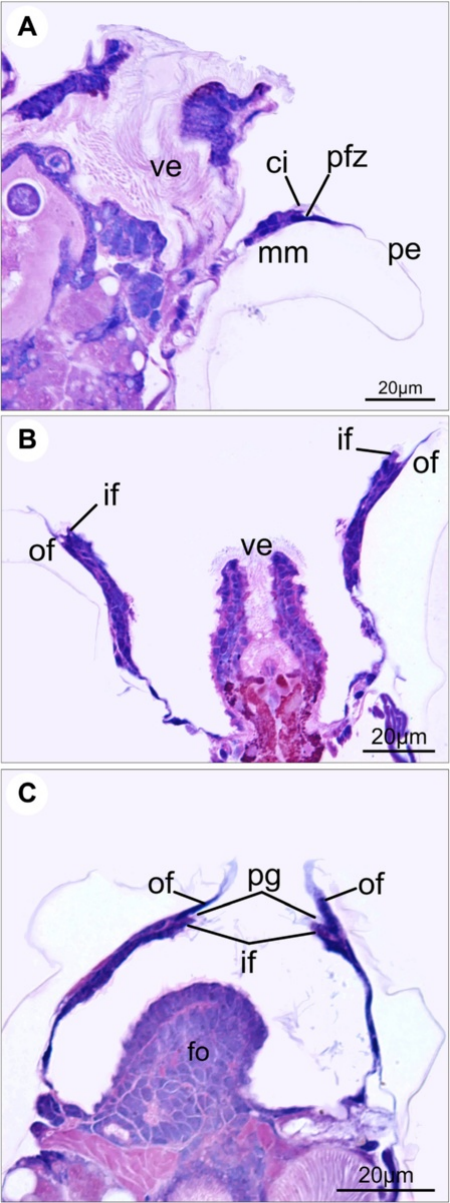
Cross sections from late pediveliger larvae of *Nodipecten nodosus.* Developmental sequence from **a**–**c** (anterior to the top). Toluidine blue and basic fuchsin. **a** Unfolded mantle margin with cilia in the periostracum-forming zone. **b** Older larva with proximal region of the mantle margin slightly projected forming the inner mantle fold. **c** Slightly older larva. Two-folded mantle margin with distinctive outer and inner folds. Abbreviations: ci, cilia; fo, foot; if, inner mantle fold; mm, mantle margin; of, outer mantle fold; pe, periostracum; pfz, periostracum-forming zone; pg, periostracal groove; ve, larval velum

In late pediveliger larvae, when the foot is completely developed and the velum gradually shrinks, another crucial modification takes place in the mantle margin. An outgrowth process gradually occurs where the ciliary row is located, adjacent to the periostracum-forming zone, resulting in a lateral evagination of the proximal region (Fig. 6a–c). This outgrowth process is responsible for the appearance of an inner pallial extension and, at the same time, for positioning the periostracum-forming zone (and respective ciliary row) at the bottom of the newly formed groove, i.e., the periostracal groove (Fig. 6c). The distal region of the mantle margin, permanently covered by the periostracum, corresponds to the outer pallial fold, while the proximal region is modified into a projection, henceforward named inner pallial fold (but see Discussion).

**Fig. 7:**
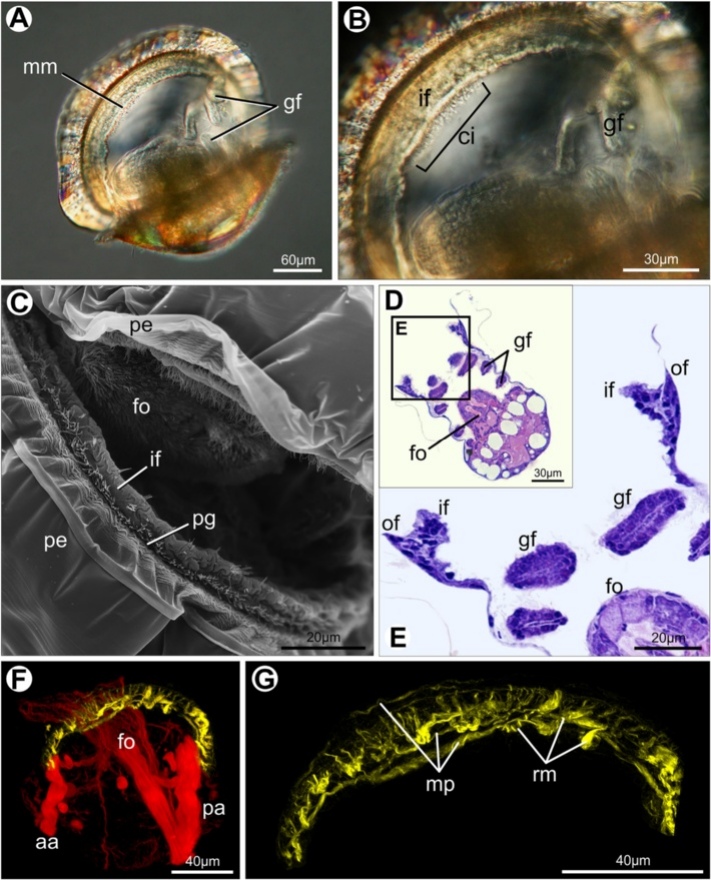
Postmetamorphic *Nodipecten nodosus* scallops. Ventrolateral view in **a**–**c** (anterior to the left), cross-section in **d** and **e** (ventral to the top and left), and lateral view in **f** and **g** (anterior to the left and ventral to the top). **a** Light micrograph with differential interference contrast. **b** Detail from the same individual showing the inner curtain-like fold, with cilia on its edge. **c** Mantle margin with inner fold and row of cilia on the periostracal groove, scanning electron micrograph (SEM). **d** Cross section of an entire specimen showing extension of the mantle and pallial cavity where gill filaments are developing. Toluidine blue and basic fuchsin (TB). **e** Detail of the same region of the inset indicated in **d**, showing the pallial cavity and the two-folded mantle margins. TB. **f** Postmetamorphic musculature stained with phalloidin and reconstructed based on confocal micrographs. Mantle muscles are presented in yellow and other muscular groups (i.e. foot retractor and adductors) are in red. **g** Detail of the pallial musculature from **f** showing retractor and margin-parallel muscles. Abbreviations: aa, anterior adductor; ci, cilia; fo, foot; gf, gill filaments; if, inner mantle fold; mm, mantle margin; mp, margin-parallel muscles; of, outer mantle fold; pa, posterior adductor; pe, periostracum; pg, periostracal groove; rm, retractor muscles

Metamorphosis results in profound changes in larval anatomy and behavior. The settled larva secretes the dissoconch, while internal organs are reorganized and rotated. The larval velum degenerates and the first gill filaments grow rapidly (Fig. 7a, b). While the larval mantle margin is inconspicuous and not visible in live specimens, the mantle margin in postlarvae is evidenced by the presence of a well-developed curtain-like inner fold bearing cilia (Fig. 7b, c). The mantle lobes become much longer, extending the free margin far from the visceral organs, providing space for the numerous developing gills within the pallial cavity (Fig. 7d, e). The pallial musculature is enlarged, bundles grow thicker, but the combined arrangement of margin-parallel bundles and retractor muscles persists (Fig. 7f, g).

**Fig. 8:**
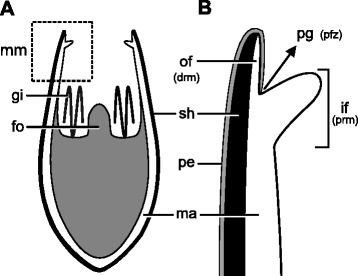
Schematic representation of the postmetamorphic mantle margin in *Nodipecten nodosus*. **a** Schematic representation of a cross-section through the animal body, ventral to the top (foot points upwards). **b** Detail of the inset indicated in **a**. The two-folded condition includes the outer and inner mantle folds, which correspond to the distal and proximal regions of the larval mantle margin, respectively. The periostracum-forming zone ends up on the bottom of a groove between the folds. Abbreviations: drm, distal region of the mantle margin; fo, foot; gi, gill; if, inner mantle fold; ma, mantle; of, outer mantle fold; pe, periostracum; pfz, periostracum-forming zone; pg, periostracal groove; prm, proximal region of the mantle margin; sh, shell

**Fig. 9:**
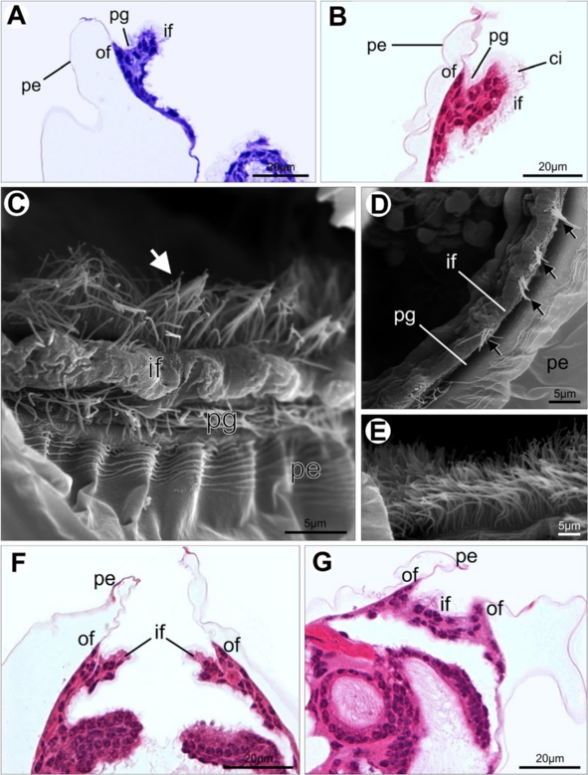
Postmetamorphic mantle margin in *Nodipecten nodosus.* Histological cross sections (**a**, **b**, **f**, **g**, ventral to the top) and scanning electron micrographs (**c**–**e**). **a** Mantle margin showing the two-folded condition with cilia on the periostracal groove. Toluidine blue and basic fuchsin. **b** Anterior region of the left mantle margin showing a developed inner fold with a dense ciliary band on the inner surface. Hematoxylin and eosin (HE). **c** Detail of the anterior region of the left mantle margin showing rows of cilia on the periostracal groove, scattered cilia on the rim of the inner fold, and dense ciliary band (arrow) on the inner surface of the inner fold. **d** Tufts of cilia (arrows) scattered along the rim of the inner mantle fold. **e** Detail of the dense ciliary band on the inner surface of the left inner fold. **f** Free edges of the mantle margin in the ventral region. HE. **g** Fused inner folds in the dorsal region; periostracal groove and outer fold remain intact. HE. Abbreviations: ci, cilia; if, inner fold; of, outer fold; pe, periostracum; pg, periostracal groove

The mantle margin in postmetamorphic scallops comprises the outer and inner folds (Fig. 8), as in late pediveligers. The outer fold is usually reduced, covered by the periostracum, while the curtain-like inner fold is pronounced (Fig. 9a, b, c). Two types of cilia arrangement are present on the inner fold. The first type, found along the rim of the inner fold, corresponds to ciliary tufts sparsely scattered over the epithelium and contains few cilia (Fig. 9d). The second type comprises a ciliary band in which long cilia are densely distributed on the inner surface of the inner fold. This latter type, however, is restricted to the anterior portion of the left mantle lobe (Figs. 7b, 9b, c, e). In live animals, intense ciliary beating from this latter type is produced in this region. In the dorsal region of postmetamorphic individuals, the inner folds are fused both anteriorly and posteriorly, the outer fold and periostracal groove remaining intact (Fig. 9f, g). Such fusion occurs near the mantle isthmus where the right and left mantle lobes are formed.

**Fig. 10:**
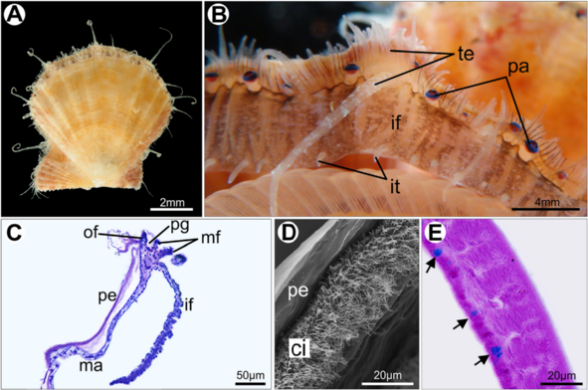
Juvenile scallops of *Nodipecten nodosus.* Mantle margin as revealed by magnifying lens (**a**, **b**), histological cross sections (**c**, **e**) and scanning electron microscopy (**d**). **a** Juvenile individual few weeks after metamorphosis. Lateral view, anterior to the left, ventral to the top. **b** Detail of the mantle margin with a large inner fold (pallial curtain or “velum”) and numerous tentacles and eyes on the middle fold; ventrolateral view, anterior to the left. **c** Three-folded condition of the mantle margin including the newly formed middle fold provided with tentacles. Toluidine blue and basic fuchsin. **d** Detail of the outer epithelium of the middle fold, showing dense cilia covering continuous with the periostracal groove. **e** Secretory cells (arrows) in the outer mantle epithelium, containing acid mucopolysaccharides stained by Alcian Blue (PAS + Alcian Blue method). Abbreviations: ci, cilia; if, inner mantle fold; it, inner fold tentacles; ma, mantle; mf, middle mantle fold; of, outer mantle fold; pa, pallial eye; pe, periostracum; pg, periostracal groove; te, middle fold tentacles

**Fig. 11:**
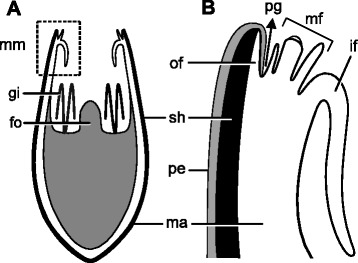
Schematic representation of the juvenile three-folded mantle margin in *Nodipecten nodosus*. **a** Schematic representation of a cross-section through the animal body, ventral to the top (foot points upwards). **b** Detail of the region indicated in the inset of **a**. The curtain-like inner mantle fold is greatly enlarged. The outer fold and the periostracal groove are similar to the postmetamorphic arrangement. The last fold to be formed, the middle one, is provided with numerous tentacles and pallial eyes. Abbreviations: fo, foot; gi, gill; if, inner mantle fold; ma, mantle; mf, middle mantle fold; mm, mantle margin; of, outer mantle fold; pe, periostracum; pg, periostracal groove; sh, shell

In juvenile individuals of *Nodipecten nodosus*, most of the adult morphological features are already established (Fig. 10a). A few weeks after metamorphosis the pallial margin comprises three folds, including the newly formed middle fold, which contains numerous eyes and tentacles (Fig. 10b, c). The scallop three-folded condition is represented in Fig. 11. The outer epithelium of the middle fold (i.e., the one facing the periostracal groove) exhibits densely distributed cilia continuous with the cilia of the periostracal groove (Fig. 10d). The inner fold, usually referred to as “velum” for pectinid bivalves, is a muscular curtain-like fold (Fig. 10c). While mucin cells were not detected in the mantle of *N. nodosus* larvae, they were observed in juvenile individuals. In those specimens, gland cells producing acidic mucopolysaccharides are spread in the outer epithelium of the mantle and outer fold, suggesting intense mucous secretion in the extra-pallial cavity, i.e., the space between the mantle and the shell (Fig. 10e).

**Fig. 12:**
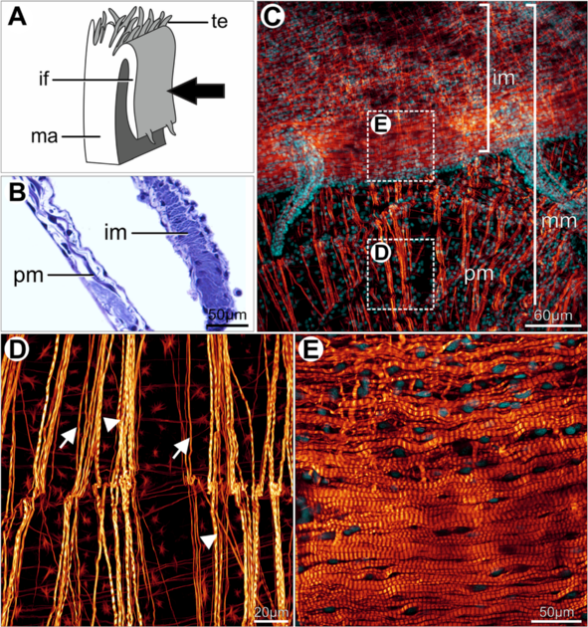
Pallial musculature in *Nodipecten nodosus* juveniles. Histological sectioning (**b**) and staining with phalloidin combined with confocal laser scanning microscopy (**c**–**e**). **a** Schematic representation of the mantle margin, ventral to the top; the arrow points to the view of figures **c**–**e**. **b** Detail of muscle fibers in the mantle and dense muscular bundles in the inner mantle fold. The plane of section is the same of figure **a**. Toluidine blue and basic fuchsin. **c** Overview of the mantle margin, including the inner mantle fold and its tentacles. Pallial muscles are radially distributed throughout the mantle margin and margin-parallel muscles are present within the inner fold. Nuclei stained in blue by DAPI; lateral view, ventral to the top. **d** Detail of the mantle margin radial musculature, as indicated in the inset of **c**, showing a combination of apparently non-striated (arrows) and striated myofibers (arrowheads). **e** Detail of the inset observed in **c** showing the arrangement of margin-parallel striated muscle fibers within the inner fold, more densely arranged in the distal region of the fold. Nuclei stained in blue by DAPI. Abbreviations: if, inner mantle fold; ma, mantle; mm, mantle margin; pm, pallial muscles; te, tentacles

In juveniles, the pallial musculature becomes evident in histological sections due to the increase of thickness and number of bundles (Fig. 12a, b). Within the mantle margin, numerous long muscles are radially distributed, with striated and apparently smooth fibers mixed in parallel bundles running from the pallial line to the mantle margin (Fig. 12c, d). Within the inner fold, besides radial muscles, densely organized striated muscle bundles running parallel to the mantle margin are present (Fig. 12b, c). These bundles are more closely arranged in the distal region of this fold (Fig. 12e).

**Fig. 13:**
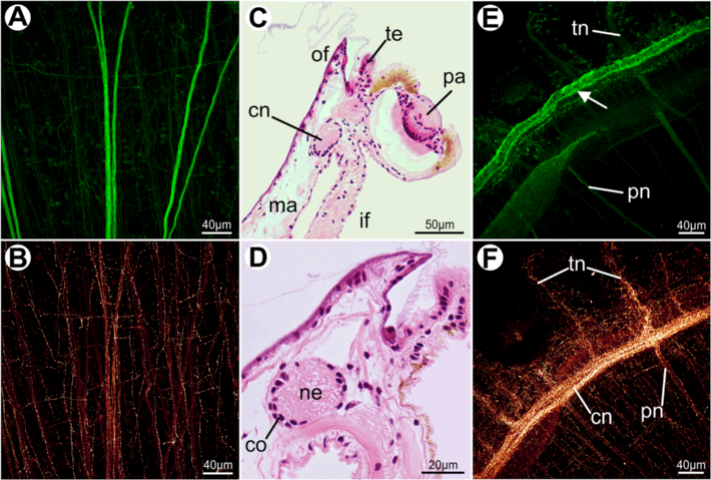
Innervation of the mantle margin in juveniles of *Nodipecten nodosus.* Histological cross sections (**c**, **d**) and immunoreactivity to serotonin (**b**, **f**) and α-tubulin (**a**, **e**) combined with confocal laser scanning microscopy. Lateral view of the mantle margin in **a**, **b**, **e** and **f** (ventral to the top), as previously indicated in the schematic representation of Fig. 12a. **a** Radial nerves, shown in green, crossing the mantle towards its distal region. **b** Serotonin-like immunoreactivity in the mantle, exhibiting a reticulate pattern of innervation. **c** Mantle margin showing the circumpallial nerve and sensorial structures such as tentacles and eyes. Hematoxylin and eosin (HE). **d** Detail of the circumpallial nerve, where a cortical region surrounds the neuropil. HE. **e** Strong α-tubulin immunoreactivity in pallial nerves reaching the distal marginal region. Arrow points to the ciliated outer surface of the middle fold. **f** Circumpallial nerve and tentacle nerves showing strong serotonin-like immunoreactivity. Abbreviations: cn, circumpallial nerve; co, cortical layer; if, inner mantle fold; ma, mantle; ne, neuropil; of, outer mantle fold; pa, pallial eye; pn, pallial nerve; te, tentacle; tn, tentacle nerve

The nervous system of the mantle margin comprises numerous radial nerves that run towards the distal region and exhibit strong immunoreactivity for α-tubulin (Fig. 13a, e). In addition, the serotonergic pallial system includes a reticulate pattern of distribution over the entire mantle margin (Fig. 13b). The very prominent circumpallial nerve is responsible for innervation of the pallial margin (Fig. 13c, d, f). Unlike other nerves, completely formed by fibrous projections combined with glia cells, the circumpallial nerve displays a typical ganglionic structure. The central portion (i.e., the neuropil) is composed exclusively by neuronal fibers, while the periphery (i.e., the cortical region) contains the cell bodies (Fig. 13d).

**Fig. 14:**
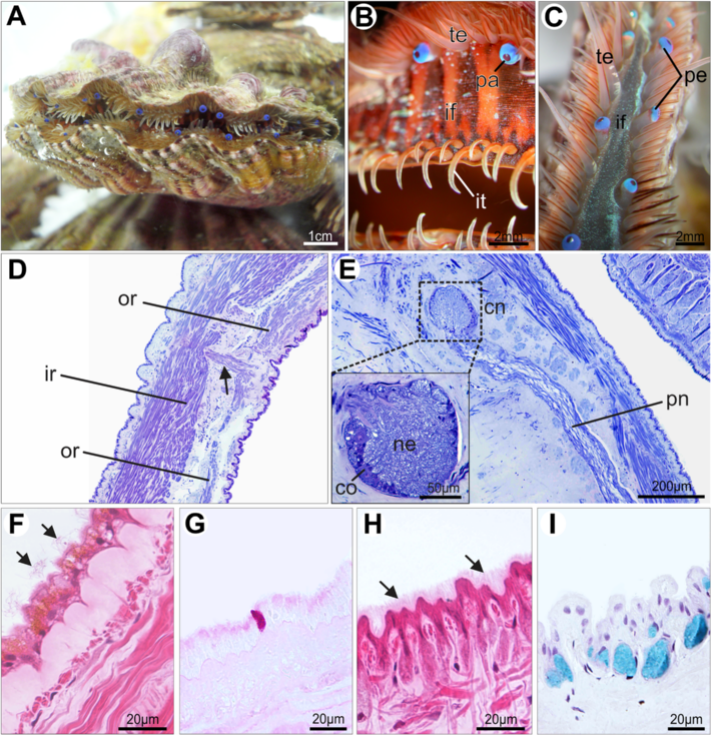
Mantle margin of adult *Nodipecten nodosus*. Mantle margin as revealed by magnifying lens (**a** and **b**, ventral view, anterior to the left; **c**, posterior view, dorsal to the top) and histological cross sections (**d**–**i**). **a** Live specimen with valves slightly opened, showing the pallial tentacles and eyes. **b** Detail of the mantle margin in an anesthetized specimen, showing the large inner mantle fold with tentacles and the middle fold with tentacles and eyes. **c** Detail of the mantle margin at the auricular region where the inner folds from both mantle lobes are fused. **d** Adult pallial musculature composed of radial bundles (divided into outer and inner groups) and transverse bundles (arrow). Ventral to the top. Toluidine blue and basic fuchsin (TB). **e** Long pallial nerve connecting to the circumpallial nerve, the cortical region and the neuropil of which are shown in detail in the inset. Ventral to the top. TB. **f** Inner mantle epithelium with cells bearing tufts of cilia (arrows). Hematoxylin and eosin (HE). **g** Detail of neutral mucopolysaccharide secretion (magenta) in a gland cell from the inner mantle epithelium. Periodic acid-Schiff stain (PAS). **h** Outer mantle epithelium with long microvilli (arrows) covering the surface. HE. **i** Subepithelial secretory cells with acid mucopolysaccharide content (light blue) in the outer mantle epithelium. Alcian blue (AB). Abbreviations: cn, circumpallial nerve; co, cortex; if, inner mantle fold; ir, inner radial muscles; it, inner fold tentacle; ma, mantle; ne, neuropil; of, outer mantle fold; or, outer radial muscles; pa, pallial eye; pn, pallial nerve; te, middle fold tentacle

Adult individuals of *Nodipecten nodosus* (Fig. 14a) display a very prominent mantle margin, with numerous tentacles, eyes, and a pigmented velum (Fig. 14b). At the auricular region (dorsal ears), the inner fold remains fused (Fig. 14c). The mantle is proportionally larger and thicker than in juveniles, and the outer fold is smaller than the remaining folds. The pallial musculature consists of large bundles of striated and smooth fibers, which form two major muscle groups that run close to the outer and inner epithelium, respectively (Fig. 14d). Small transverse muscles are also present (Fig. 14d). Embedded within the connective tissue, pallial nerves reach the circumpallial nerve at the level of the pallial folds (Fig. 14e). The circumpallial nerve preserves its ganglionic organization, i.e., with neuronal bodies distributed at the periphery and numerous axons merged at the core (Fig. 14e).

**Fig. 15:**
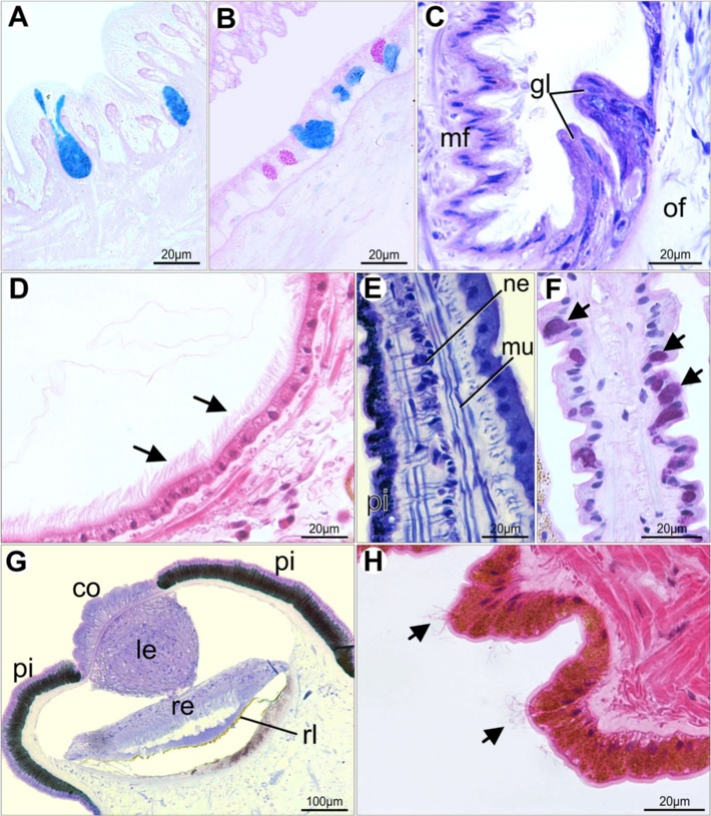
Histology of the mantle folds of adult *Nodipecten nodosus*. **a** Acid mucopolysaccharide-secreting cells spread over the outer mantle epithelium. AB. **b** Two distinct types of secretory cells in the inner epithelium of the outer mantle fold: Alcian Blue-positive (acid mucopolysaccharides) and PAS-positive (neutral mucopolysaccharides; magenta color) cells. **c** Detail of the periostracal groove with two glandular folds responsible for periostracum secretion at the bottom of the groove. Toluidine blue and basic fuchsin (TB). **d** Ciliated epithelium (arrow) of the outer surface of the middle mantle fold. Hematoxylin and eosin (HE). **e** Middle fold tentacle displaying muscle and nervous fibers and a pigmented epithelium. TB. **f** PAS-positive mucous cells (arrows) in the middle fold tentacle (nuclei stained with hematoxylin). **g** Section through the pallial eyes showing its general structure. TB. **h** Outer epithelium of the inner mantle fold with sparse tufts of cilia (arrows). HE. Abbreviations: co, cornea; gl, periostracal glands; le, lens; mf, middle mantle fold; mu, tentacle muscles; ne, tentacle nerve; of, outer mantle fold; pg, periostracal groove; pi, pigmented epithelium; re, retina; rl, reflector layer; te, tentacles

Simple, cuboidal cells are found in the inner epidermis of the outer fold, in the middle fold, and in the inner pallial epithelium. Simple, columnar cells are common in the inner fold, in the outer pallial epithelium, as well as in the outer epithelium of the outer fold. The adult inner mantle margin epidermis is covered by sparsely distributed tufts of cilia (Fig. 14f) and few epithelial mucous cells with neutral mucopolysaccharide content (Fig. 14g). The outer mantle margin epithelium differs from the inner one by the presence of short microvilli (Fig. 14h) and numerous secretory cells containing exclusively acidic mucopolysaccharide vesicles (Fig. 14i). This condition remains the same at the outer epidermis of the outer fold (Fig. 15a). However, the inner epithelium of the outer fold possesses two types of secretory cells related to acidic and neutral mucopolysaccharide secretion (Fig. 15b). At the periostracal groove, a pair of glandular folds is responsible for periostracum secretion (Fig. 15c).

**Fig. 16:**
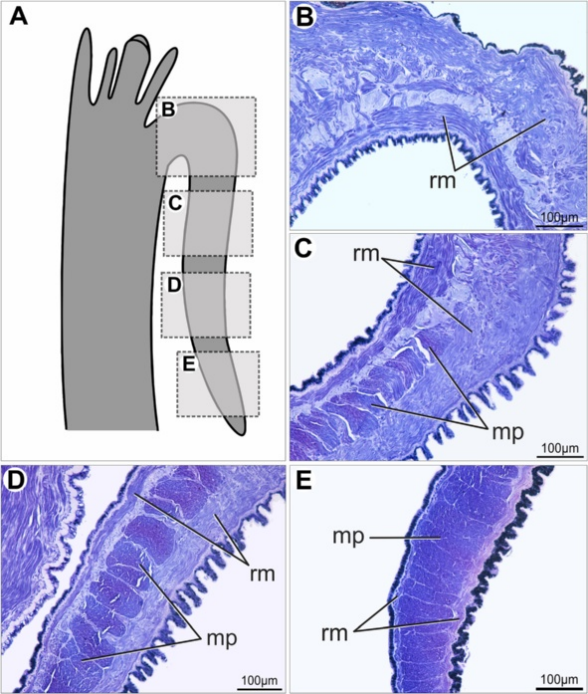
Histological sections through the inner mantle fold of *Nodipecten nodosus*. Sequence of sections (B–E) from proximal to distal region, showing gradual increase in margin-parallel musculature towards distal. Toluidine blue and basic fuchsin. **a** Schematic representation of the mantle margin, specifying the regions from the inner mantle fold that correspond to the sections seen in B–E. Ventral to the top. **b** Radial muscles from the mantle extend into the base of the inner fold. **c** At intermediate levels of the inner fold, there is a combination of radial and margin-parallel muscles. **d** Towards the margin, the connective tissue is reduced and margin parallel muscles increase in volume. **e** In the distal region, the radial muscles are compressed and margin-parallel muscles predominate. Abbreviations: rm, radial muscles; mp, margin parallel muscles

The adult middle fold epithelium is intensely ciliated on its outer surface (Fig. 15d). The middle fold tentacles contain mucous and pigment cells (Fig. 15e, f), while the pallial eyes display the typical scallop ocular components (Fig. 15g). The inner fold is the largest structure of the adult pallial margin, bearing small tentacles at its most distal region (Fig. 14b), and sparse tufts of cilia on its surface (Fig. 15h). The inner fold musculature becomes more prominent and regionalized in adults (Fig. 16a–e). Radial muscles emerge from the pallial musculature and extend through the entire fold (Fig. 16b), running from the base of the velum to the marginal end (Fig. 16b–e). The margin-parallel muscles seen in juveniles are now organized in bundles that become more numerous and larger as they approach the middle and distal portions (Fig. 16d, e). During this gradual transition, the radial musculature is laterally compressed against the base of the epithelium, providing space for the margin-parallel fibers (Fig. 16e).

## Discussion

### Larval mantle folds and periostracum-forming zone

The larval mantle margin in *Nodipecten nodosus* is unfolded, comprising a single projection divided into distal and proximal regions by the periostracum-forming zone. Nevertheless, many authors have adopted the term “fold” to designate such division in early larval stages, which may have led to possible misinterpretations of larval morphology, particularly concerning mantle margin development. Two folds were reported at the mantle rim in larvae of *Pecten maximus*: the “outer fold” being covered by the periostracum, and the epithelium of the “inner fold” being exposed and covered by short microvilli [26]. However, the schematic representation of the mantle margin in *P. maximus* [26] exhibits no folds, but proximal and distal regions, quite similar to the larval pallial organization of *N. nodosus* described herein. Other similar examples include the mantle margin of *Crassostrea virginica* [53], *Cardium edule* [54], *Nucula delphinodonta* [55], *Lasaea adansonii* [56], and *Ostrea edulis* [37], where the figures (illustrations or photographs based on light or electron microscopy) provided by the authors all clearly show an unfolded larval margin. Considering the results obtained in the present study combined with the above-mentioned information, it seems more suitable to use the terms “distal” and “proximal” regions rather than “fold” to designate specific areas of the larval mantle margin.

Although it is assumed that the presence of two pallial folds corresponds to the typical condition of bivalve larvae [35], most of the available information for the larval mantle margin is restricted to observations of a single larval stage, generally with no data on previous stages (e.g. [35]). In a developmental perspective, this could suggest that early mantle traits are unknown for some species. Therefore, it is reasonable to consider that, at least for some bivalves such as scallops, the larval mantle margin may exhibit an unfolded step in early development, with subsequent division into two mantle folds. However, the early mantle margin in other bivalve species undoubtedly does show a folded condition, thus highlighting the importance of more comparative and detailed studies to clarify such issues.

In *Nodipecten nodosus*, the larval periostracum begins as a wrinkled band before covering the distal margin and shell, a condition also detected for *Ostrea edulis* [37], and referred as “convoluted periostracum” [35]. This organic sheath is comprised by three layers formed within a groove between the outer and inner folds of bivalve larvae [35]. However, periostracum secretion commences before the mantle folds are developed, i.e., before a groove is present. In this case, the secretory activity occurs on a line at the distal region of the inner mantle margin epithelium [26, 56], as confirmed by the TEM observations presented herein for *N. nodosus*. The term “periostracum-forming zone” was proposed to designate the site where the organic sheath is produced in the unfolded mantle margin of *Lasaea adansonii* larvae [56], and we agree with the appropriateness of this term.

### Mantle margin development

#### General development of the mantle margin

**Fig. 17:**
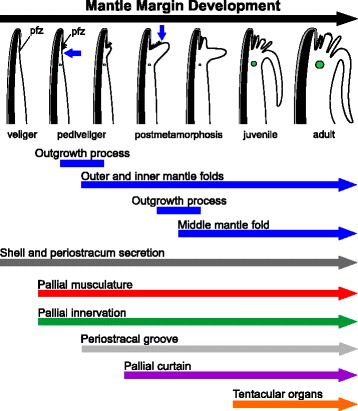
Schematic representation of the development of the mantle margin in *Nodipecten nodosus*. Processes are depicted in different colors for the same developmental sequence; blue arrows on the drawings indicate the point of origin of the inner and middle folds; green circles indicate the circumpallial nerve. All images are in cross-section view with the anterior to the top in veliger and pediveliger stages, and ventral to the top in postmetamorphic stages. Two outgrowth processes are responsible for mantle fold formation (blue). The first one occurs in late pediveligers, forming the inner and outer mantle folds. After this process, periostracum secretion is situated in a groove between the folds. The second outgrowth process takes place after metamorphosis, giving rise to the middle mantle fold from the inner surface of the inner fold. Periostracum and shell secretion (grey) is continuous throughout development since the onset of shell field formation in the trochophore stage. Both muscle (red) and nervous systems (green) are initially formed by the early pediveliger stage, and further modifications are involved during fold development. See text for more information. Abbreviation: pfz, periostracum-forming zone

A hypothesis for the development of the pectinid mantle margin is summarized in Fig. 17 and is proposed based on the data obtained herein for *Nodipecten nodosus* combined with revisited information from the literature (e.g. [26, 28, 35, 37, 53, 54, 56, 57]). Even though the developmental sequence includes some unique characters from scallops, such as the pallial eyes from the middle fold, the morphogenesis is represented in order to provide general insights into mantle margin development in the Pteriomorphia.

The bivalve mantle is formed shortly after the appearance of the shell field on the dorsal surface of late trochophore larvae [31, 38]. The epithelium of the shell gland then extends, giving rise to the mantle which will permanently underlie the valves [36, 58]. In scallops, the veliger exhibits an unfolded mantle margin, i.e., a single projection beneath the shell margin with no distinct folds. The periostracum formation zone (PFZ) marks the division between the distal region of the mantle margin, which is permanently covered by the periostracum, and the proximal region. During larval development, the transition to the pediveliger stage is marked by the emergence of a row of cilia in the proximal region, adjacent to the PFZ. In addition, the pallial musculature arises, as does the mantle innervation. The unfolded condition is conserved until slightly before metamorphosis, when the first of two major outgrowth processes takes place. An evagination of the proximal region near the row of cilia produces the inner mantle fold, the distal region now corresponding to the outer fold. Consequently, the PFZ is confined within a groove, and the row of cilia, previously present on the surface of the proximal mantle region, comes to lie on the outer surface of the inner mantle fold. Metamorphosis produces a variety of anatomical changes, the inner fold becoming a prominent pallial curtain-like fold. Also, at this stage the second outgrowth process occurs, forming the middle fold through an evagination at the base of the outer surface of the inner fold. Consequently, the ciliated epithelium of the periostracal groove is now located on the outer surface of the middle fold. Once the three-folded condition is achieved, further development includes the growth of sensorial organs (middle fold tentacles and eyes first appearing at the juvenile stage) and further growth of the pallial curtain.

Alternatively, the presence of cilia on the outer epithelium of the middle fold in juveniles and adults could be interpreted as a condition that originates before metamorphosis and is preserved during subsequent development. In this case, the middle mantle fold would form earlier, while the inner fold would emerge later as a projection from its inner surface, after metamorphosis. However, some lines of evidence support the first hypothesis (earlier formation of the inner fold; Fig. 17): 1) the first outgrowth process generates a curtain-like fold, which is morphologically and functionally similar to the inner fold of later stages; 2) pallial tentacular structures emerge only after metamorphosis, which is in accordance with a late formation of the middle mantle fold (that bears these structures); 3) the dorsal fusion of pallial folds is already present shortly after metamorphosis, when a third fold is not yet present. Such fusion corresponds to the merging of both inner folds at the auricular zone of juvenile and adult scallops, thus also supporting the early emergence of this pallial fold.

#### Implications for hypotheses concerning mantle fold development

The present investigation on the development of the mantle margin in *Nodipecten nodosus* casts new questions on old issues concerning the anatomy and development of bivalves. For instance, an unfolded mantle margin is proposed as a possible condition, at least for some bivalves. Whereas two folds are achieved in late pediveliger larvae of *N. nodosus*, apparently such a condition may occur early in other bivalve species (e.g. [28, 35]). In addition, the groups of cilia scattered on the larval inner mantle fold of some bivalves (e.g. [35, 37]) only arise in scallops after metamorphosis. So, despite common pallial features in those bivalves, the timing of the emergence of cilia and mantle folds seems to vary. This could be explained by distinct rates of differentiation during ontogeny [59, 60], leading to heterochronic alterations across the Bivalvia. Heterochrony has been suggested as the reason for modifications in developmental time of bivalve larvae and for significant anatomical and evolutionary changes [61, 62].

In adult Arcoida, the outer mantle fold is usually subdivided into two distinct folds, while the remaining folds are more variable. While species of *Arca* and *Glycymeris* exhibit no middle fold [14], the inner fold in *Philobrya, Bathyarca*, *Barbatia,* and *Trisidos* is duplicated, showing variation in size and shape [63]. Based on epithelial differences observed in the single inner fold of *Arca noae*, it was suggested that both middle and inner folds are present in ark clams, although they are combined into one single fold [15]. In addition, the two-folded mantle margin of adults of some arks [14, 63] and the presence of two folds in oyster pediveligers [37] contributed to suppose that the outer surface of the inner mantle fold would be the putative middle fold, not ontogenetically differentiated into a distinctive structure [15]. The present investigation with *Nodipecten nodosus* has provided the first developmental evidence to support the origin of the middle fold from the outer surface of the inner fold, reinforcing this previous hypothesis [15].

The two-folded arrangement has been claimed to be a primitive condition in the Bivalvia [14, 15]. There are, however, few comparative studies on the bivalve mantle margin, particularly in Pteriomorphia, and none of them was analyzed under a phylogenetic perspective, which prevents further inferences on character evolution. We agree that analysis of developmental sequence make no a priori assumptions on the conservation of developmental stages or about how sequence should evolve [59], so we are not able to ascertain if the two-folded mantle margin is a primitive condition or a derived trait of some species (e.g., in which the middle fold would not be differentiated). Identifying homologous characters in the mantle margin is a challenging issue and represents a central point to the understanding of molluscan mantle evolution. More comparative data are still necessary, as well as developmental evidence for other taxa, to integrate hypotheses of homologies concerning mantle folds and pallial structures across the Bivalvia.

### Functional ontogeny and morphology of the mantle margin

#### Larval mantle margin anatomy

The covering of the inner epithelium of the mantle margin by microvilli seen in *Nodipecten nodosus* was previously observed in *Pecten maximus* [26] and *Ostrea edulis* [35, 37]. The presence of cilia has been reported for the larval mantle margin of several bivalves, but this information is almost always imprecise. For instance, cilia were described along the pallial margin of veligers of the scallop *Argopecten irradians*, but no details about type or distribution were provided [64]. In *N. nodosus*, cilia are not present until the pediveliger stage, when a row of long cilia is formed adjacent to the PFZ. Five ciliary types are present on the mantle margin of *P. maximus* larvae [26]. Type 1 represents few cilia located in small depressions; type 2 is a row of long cilia in line, close to the distal region of the edge; type 3 includes tufts of numerous cilia scattered on the posterior margin; type 4 is a line of single cilia around the posterior edge; and type 5 is a row of short cilia in a protuberance dorsal to the anus. Even though not all these ciliary types were detected in larvae of *N. nodosus*, the row of cilia adjacent to the periostracum-forming zone likely matches type 2.

When the mantle margin becomes two-folded in late pediveligers of *Nodipecten nodosus*, new ciliary groups arise, and after metamorphosis they become more prominent. Within the periostracal groove, dense rows of cilia are distributed on the outer surface of the inner fold. Such a ciliary band was also detected in *Ostrea edulis* [35]. Epithelial cells containing several electron-lucent vesicles were found in *Pecten maximus*, close to the ciliated cells at the PFZ [26]. In addition, great abundance of rough endoplasmic reticulum was detected in mantle margin cells of *Crassostrea virginica* [53]. In general, these ultrastructural characteristics seem in accordance with the present data for *N. nodosus*. On the edge of the inner fold of postmetamorphic *N. nodosus*, tufts of cilia are scattered along the margin, exactly like those classified as type b in pediveligers of *Argopecten purpuratus* [57], and type 3 in *P. maximus* [26]. Long cilia, similar to those present in the ciliary band from the inner surface of the inner mantle fold of postmetamorphic *N. nodosus*, also occur in tufts along the inner larval fold of *Mytilus edulis* [65], *O. edulis* [35], and *Pinna carnea* [66]. However, no cilia were detected in the mantle margin of *Pandora inaequivalvis* larvae prior to or after metamorphosis [67]. Tufts of cilia in the inner fold of oyster pediveligers are related to sensory cells, while the wide ciliary band may display cleansing functions [35]. However, in the case of *N. nodosus* postlarvae, the wide ciliary band is restricted to the anterior portion of the inner fold, within the inhalant region of the pallial cavity. In live postlarvae, those cilia exhibit intense beating, which may suggest a potential contribution to water flow into the mantle cavity instead of a sensory function.

The larval anatomy of the bivalve pallial musculature and nervous system remains largely unknown. Some muscle fibers were described for the inner fold of some Pinnidae species [66], but a detailed comparison with the present data is prevented, since no image was provided in that study. Smooth fibers are supposed to be present near the mantle folds of *P. maximus* based on larval descriptions [26]. In pediveligers of *O. edulis*, radial muscles were supposed to be present in the larval mantle margin based on mantle contractions of live specimens [37]. The present investigation of *Nodipecten nodosus* provides clear evidence for the emergence of pallial musculature during the pediveliger stage, including retractor and margin-parallel bundles. The formation of pallial muscles in mid-stage larvae was also observed in *Lasaea adansonii*, where both muscle types are formed by smooth fibers and spread over the mantle margin [56]. Regarding pallial innervation, the mantle of *N. nodosus* seems to become assisted by the peripheral nervous system only by the pediveliger stage, when serotonin-like projections extend into the mantle, forming a nerve that runs parallel to the margin. Besides the possible presence of neuronal cells adjacent to the mantle margin [40], a serotonin-like nerve was detected running along the mantle in *Mytilus edulis* pediveligers, and catecholaminergic cells in *Placopecten magellanicus* [68].

#### Postmetamorphic mantle margin anatomy

The mantle of adult bivalves has been extensively studied, mainly regarding aspects of shell secretion and siphon anatomy (e.g. [7, 12, 32, 69, 70]). The bivalve mantle epithelium generally comprises cuboidal to columnar cells, with numerous secretory cells spread across both surfaces. The mantle lobes may exhibit free margins partially united in specific regions, as commonly observed in several protobranch and pteriomorphian groups, as well as different degrees of marginal fusion, including a variety of siphons, such as those present in infaunal bivalves [6, 16, 62]. In the auricular region of *Nodipecten nodosus*, the inner folds from both lobes are dorsally fused, characterizing a very restricted fusion in contrast to the wide free ventral margins. In general, reduced or entire absence of fusion are common features in the mantle margin of epifaunal bivalves from the Pteriomorphia clade [66].

While the outer mantle fold remains unaltered and covered by the periostracum after metamorphosis, the inner fold of *Nodipecten nodosus* becomes greatly pronounced into the typical curtain-like fold (“velum”) observed in grown scallops [71]. In this respect, “pallial curtain” is the term applied to the hypertrophied marginal projection of the inner fold that controls the passage of water into and out of the pallial cavity [72]. Pallial curtains are also found in Ostreidae and Pteridae, where the inner mantle fold is enlarged [6].

As demonstrated herein, the middle fold emerges after metamorphosis, giving rise to tentacles and eyes in *Nodipecten nodosus* and establishing the three-folded pattern. The typical functions concerning the bivalve mantle fold are present in the Pectinidae, including a secretory outer mantle fold, a sensorial middle fold, and a muscular inner fold [6, 7]. The concentration of chemical, mechanical, and optical receptors in the middle mantle fold have stimulated extensive studies on their functional properties. The pallial eyes, remarkable by their complexity and debatable evolutionary significance, have been deeply studied [45, 47, 49, 73–75], and recent molecular investigations have cast some light on the origin and diversification of such organs [76, 77].

The late larval musculature of the mantle of *Nodipecten nodosus* seems to be preserved after metamorphosis. Notwithstanding, little is known about the anatomy of the bivalve mantle musculature shortly after metamorphosis. In *Mytilus trossulus*, the postlarval muscles of the mantle margin comprise margin-parallel bundles that emerge during the pediveliger stage [78]. A rapid development of mantle retractor muscles was also observed after metamorphosis in pinnids [66], but it is possible that mantle muscles were present in late larvae that were overlooked due to methodological limitations. The emergence of the pallial musculature in *N. nodosus* pediveligers indicates the possibility of effective retraction of the mantle margin into the mantle cavity, which has already been suggested for *Ostrea edulis* [37].

In *Nodipecten nodosus*, the inner fold is the most muscular region of the mantle margin, being composed mainly of numerous margin-parallel bundles of striated fibers as well as mantle retractors. In contrast, striated muscles are not present in mussels and other bivalves after metamorphosis, resulting in an entire muscular system organized by smooth myofibers [79].

The innervation of the mantle margin in adult scallops is relatively well-known for some species, the studies covering several anatomical and physiological topics [44, 71, 73, 74, 80]. Nevertheless, little information is available on its development. The circumpallial nerve is the most significant nerve in the scallop pallial margin. It runs parallel alongside its entire extension and is responsible for the innervation of the pallial folds and organs [71]. The anterior dorsal portion of the nerve is formed by fibers from the cerebral-ganglia, while the ventral and posterior regions of the mantle margin are innervated by long fibers from the visceral ganglion [73, 81]. Strong serotonergic immunoreactivity was detected in the circumpallial nerve of juvenile *Nodipecten nodosus*, as well as in projections towards mantle organs. Considering that these characteristics were also detected in the nervous system of pediveligers, it seems reasonable to conclude that the nerve along the larval mantle margin is, in fact, the circumpallial nerve (formed, therefore, even before marginal folding). The development of this specific nerve along the mantle margin may contribute to the innervation of this region, allowing for sensory functions and muscular control. Serotonin is known to play a vital role in regulation of cilia activity in bivalve and other molluscan larvae [82–84] . Such information is relevant to discuss hypotheses of the early emergence and subsequent development of neuronal activity in the pallial margin. The presence of a neuropil and a cortex in the circumpallial nerve is regarded by some authors as an anatomical evidence for the crucial role of this nerve in the mantle margin, which was even considered a marginal ganglion [81]. Finally, larval development of mantle muscles and innervation demonstrate how anatomical organization anticipates fold establishment during mantle margin morphogenesis. After metamorphosis, further modifications in both systems are deeply associated with specialization of the mantle folds and their associated structures.

#### Secretory roles

Shell secretion is generally regarded to be the main function of the mantle, which exhibits different secretory cells on the outer epithelium adjacent to the outer fold (e.g. [9, 85–87]. Biomineralization has been extensively studied in bivalve molluscs, including mechanisms of calcification and shell microstructure (e.g. [8, 88]). During development, shell formation is initiated at the late trochophore stage, when mantle gland cells are involved in secretion of the larval prodissoconch I and II, and subsequently, the postmetamorphic dissoconch [32, 33]. Apart from shell secretion, functional studies on the larval mantle margin are very scarce, although some efforts have been made to elucidate the roles of chitin secretion in bivalve larvae [89]. PAS-reactive vesicles and acidic mucopolysaccharide-protein complex content were detected in cells from the outer and inner mantle folds of *Ostrea edulis* pediveligers [35]. Although the histochemical techniques applied to *Nodipecten nodosus* have provided no evidence for such secretory roles at least until metamorphosis, secretory vesicles were detected in pediveligers by transmission electron microscopy studies. Further work is still necessary to investigate the developmental origin and possible changes in mantle secretory activity during development.

In juvenile and adults of *Nodipecten nodosus*, secretory cells are present in all three pallial folds, as well as in the remaining inner and outer mantle epithelia. The columnar cells of the outer surface of the outer fold gradually become the cuboidal epithelium observed on the inner surface of the same fold and in the rest of the mantle. Reduction of mantle epithelial thickness throughout the outer fold was reported for other bivalves, such as *Mytilus edulis*, *Cardium edule*, *Nucula sulcata* [90], and *Gomphina veneriformis* [91], and related to possible differences in secretory activities [92]. Similar to the gland cells observed in the outer mantle fold of *N. nodosus*, positive reaction to PAS and Alcian Blue was found in secretory cells from the outer fold of *Pinctada fulcata* [93] and *Cerastoderma edule* [94], suggesting intense secretion of neutral and acidic mucopolyssacharides in this region. In the extra-pallial cavity of the outer mantle fold, the calcified shell is deposited in an organic matrix formed by proteins, mucopolyssacharides, glycoproteins, and lipids [9, 85, 92]. The importance of the organic matrix for shell formation may explain the role of the several mucous-secreting cells concentrated in the outer mantle epithelium observed in these bivalves. Additionally, it has also been suggested that the products of mucous cells in this region may be concerned with mantle lubrication [86, 92]. Mantle secretory cells of *Pinctada margaritifera* exhibit mucous or granular content in all three mantle folds, although the number of cells responding to PAS-Alcian blue decreases in the middle one [95], a condition also observed in *C. edule* [94]. In contrast, numerous cells secreting acidic mucopolysaccharides were detected in the middle fold tentacles of *N. nodosus*. Although PAS-positive and Alcian Blue-positive gland cells are present in both middle and outer folds, we observed some differences within each group when applying hematoxylin to stain nuclei (different purple to bluish tones), suggesting that more than one type of mucous secretion is present.

In several bivalves, including scallops, the inner mantle fold and the inner pallial epithelia commonly display mucous cells, usually associated with ciliated cells [42, 94–96]. Cilia and mucocyte distribution on the inner mantle epidermis of *Nodipecten nodosus* are in accordance with previous observations on *Placopecten magellanicus*, where mantle cilia are suspected not to contribute much to the water flow or particle transportation inside the mantle cavity due to their sparse distribution [96]. Furthermore, the high viscosity of the acidic mucopolysaccharides secretions in the scallop mantle epithelium, in addition to the absence of a specialized mantle ciliary transportation, suggest that this mucous secretion might facilitate the passage of water over the mantle surface [42, 97, 98].

Taken together, within an evolutionary context, several issues concerning the development and diversification of bivalve mantle folds remain unclear. The evolution of the bivalve mantle margin might have been associated with the emergence of new functions, new pallial structures, and fold specialization [14]. Nevertheless, general hypotheses concerning both phylogenetic and developmental diversification of the bivalve mantle margin are still challenging. Although our current knowledge of mantle margin diversity is very fragmentary, the plasticity of this region is obvious [15]. Future investigations on the bivalve mantle margin should aim at identifying and testing hypotheses of homologies for mantle folds and pallial structures across bivalve taxa. Our study provides a developmental background for large-scale comparative evodevo analyses into these issues. The mantle margin is a complex system, and its evolution should be regarded as an anatomical and developmental innovation which, under varying selective forces, has led to considerable diversification of form and function.

## Conclusions

Our results support a previous hypothesis [15] concerning the origin of the middle mantle fold from the outer surface of the inner fold, and that an initially unfolded condition of the mantle margin may represent a common feature for at least some bivalve mollusks. In addition, our study highlights the importance of the larval period for mantle margin morphogenesis, during which the first outgrowth process (by which the outer and inner folds are formed) and the emergence of the pallial musculature and the innervation of the mantle occur. Consequently, both larval and postmetamorphic changes are crucial for the establishment of the later three-folded condition seen in juveniles and adult bivalves.

## Methods

### Animals

Specimens of *Nodipecten nodosus* at different developmental stages were obtained from the scallop farm *Institute of Eco-Development from Baía da Ilha Grande* (IED-BIG), Rio de Janeiro, Brazil. Veligers (about 100 μm length) correspond to larvae 2 weeks after fertilization, while pediveligers (around 150 μm length) were 3 weeks old and settled individuals (about 400 μm length) reached metamorphosis slightly before the fourth week. Juveniles (maximum of 4 mm length), around a few weeks after metamorphosis, and adult mature individuals (about 8 cm length) were obtained as well. The specimens were removed from artificial hatcheries and, in case of larvae, observed under the light microscope to check if they exhibited healthy morphology and behavior. Then, samples were anesthetized by gradual addition of drops of 7.5 % MgCl_2_ for 2 h prior to fixation.

### Histological procedures

Specimens were fixed for 3 h at 4 °C in a modified Karnovsky solution (2 % paraformaldehyde + 2.5 % glutaraldehyde in 0.1 M sodium cacodylate buffer at pH 7.4 and 1 Osm adjusted with sucrose [99]). Larvae, postlarvae and juveniles were decalcified for 12 h at room temperature in 3 % ascorbic acid in distilled water, while adult specimens were dissected after anesthesia to remove fragments from the pallial margin. Then, specimens were dehydrated in a graded ethanol series and embedded in glycol-methacrylate resin (Leica Historesin Kit). Serial sections of 2–3 μm were produced on a Leica RM2255 microtome (Leica, Wetzlar, Germany) and stained with hematoxylin and eosin (HE) or toluidine blue and basic fuchsin (TB). In order to evidence possible secretory cells in the mantle, histochemical methods were applied using periodic acid-Schiff stain (PAS) and Alcian blue (AB) for neutral and acid mucopolysaccharide staining, respectively. Digital images were captured using a Nikon eclipse 80i microscope equipped with a Nikon DS-Ri1 camera (Nikon Instech Co. Ltd, Kawasaki, Japan).

### Scanning and transmission electron microscopy

For scanning electron microscopy (SEM), samples were fixed in modified Karnovsky solution. Post-fixation was performed for 30 min in 1 % OsO_4_ in buffer solution (sodium cacodylate buffer at pH 7.4), followed by 15 min in 1 % tannic acid in buffer solution and additional 15 min in fresh solution of 1 % OsO_4_ at 4 °C. Then, specimens were decalcified as previously described for histological procedures and dehydrated in a graded ethanol series. Samples were critical point dried using CO_2_ as intermediate in a Balzers CPD 030 (Electron Microscopy Sciences, EUA), mounted on stubs, coated with gold in a Balzers SCD 050 sputter coater (Electron Microscopy Sciences, EUA), and observed in a Zeiss DSM 940 (Carl Zeiss, Oberkochen, Germany). For transmission electron microscopy (TEM), post-fixation was performed for 1 h in 1 % OsO_4_ in buffer solution. Larval samples were embedded in Epoxy resin; ultrathin sections (50–70 nm) were cut using a Leica Ultracut UCT microtome (Leica, Illinois, USA), mounted on copper slot-grids, contrasted with uranyl acetate and lead citrate, and analyzed using a Zeiss EM 900 electron microscope (Carl Zeiss, Oberkochen, Germany).

### Immunocytochemistry, confocal laser scanning microscopy and 3D reconstruction

Specimens were fixed in 4 % paraformaldehyde in 0.1 M phosphate buffered (PB) for 1 h, followed by four rinses with buffer solution. Until further preparation, all samples were stored in 0.1 M PB containing 0.1 % NaN_3_ at 4 °C. Prior to staining procedures, larval and postmetamorphic individuals were decalcified in 0.05 M EGTA for 1 h. Juvenile specimens were dissected in order to remove small fragments of the mantle margin for confocal laser scanning microscopy (CLSM).

For F-actin staining, specimens were permeabilized in PB containing 2 % Triton-X 100 (PBT) overnight and then incubated in a 1:40 dilution of Alexa Fluor 488 phalloidin (Molecular Probes) in PBT for 24 h at room temperature in the dark. For neuronal staining, larval specimens were incubated in 6 % normal goat serum in PBT (block-PBT) overnight at room temperature. Subsequently, primary antibodies (e.g., anti-serotonin raised in rabbit and anti-α-tubulin raised in mouse) were applied at a concentration of 1:400 in block-PBT for 24 h. Then, specimens were rinsed several times in block-PBT prior to application of a secondary fluorochrome-conjugated antibody (goat anti-rabbit Alexa Fluor 488 and goat anti-mouse Alexa Flour 633, Molecular Probes) in block-PBT at a concentration of 1:200 for 24 h in the dark. Nuclei were stained by adding a 1 μl drop of 4′, 6-diamidino-2-phenylindole (DAPI, Invitrogen) in conjunction with secondary antibody or phalloidin incubation. Then, all samples were washed three times in PBS for about 30 min and mounted in Fluoromount G (Southern-Biotech, Birmingham, EUA) on standard microscope slides which were stored in the freezer prior to analysis. Analysis and image acquisition were performed on a Leica TCS SP5 II confocal laser scanning microscope equipped with the software Leica Application Suite Advanced Fluorescence (LAS AF), Version 2.6.0 (Leica Microsystems, Wetzlar, Germany). Confocal image stacks were recorded with 0.3 μm step size along the z-axis and digitally merged as maximum intensity projections. 3D reconstructions were created from selected confocal stacks using the imaging software Imaris, Version 4.1 (Bitplane, Zürich, Switzerland).

## Abbreviations

AB: Alcian blue
CLSM: Confocal laser scanning microscopy
block-PBT: Phosphate buffer solution containing Triton-X 100 and 6 % normal goat serum
HE: Hematoxylin and eosin
PAS: Periodic acid-Schiff stain
PB: Phosphate buffer solution
PBT: Phosphate buffer solution containing Triton-X 100
PFZ: Periostracum-forming zone
SEM: Scanning electron microscopy
TB: Toluidine blue and basic fuchsin
TEM: Transmission electron microscopy
